# Association Between Brain Substructure Dose and Cognitive Outcomes in Children With Medulloblastoma Treated on SJMB03: A Step Toward Substructure-Informed Planning

**DOI:** 10.1200/JCO.21.01480

**Published:** 2021-10-29

**Authors:** Sahaja Acharya, Yian Guo, Tushar Patni, Yimei Li, Chuang Wang, Melissa Gargone, Jason M. Ashford, Lydia Wilson, Austin Faught, Wilburn E. Reddick, Zoltan Patay, Amar Gajjar, Heather M. Conklin, Thomas E. Merchant

**Affiliations:** ^1^ Department of Radiation Oncology, St Jude Children's Research Hospital, Memphis, TN; ^2^ Department of Radiation Oncology and Molecular Radiation Sciences, Johns Hopkins Medicine, Baltimore, MD; ^3^ Department of Biostatistics, St Jude Children's Research Hospital, Memphis, TN; ^4^ Department of Psychology, St Jude Children's Research Hospital, Memphis, TN; ^5^ Department of Diagnostic Imaging, St Jude Children's Research Hospital, Memphis, TN; ^6^ Division of Neuro-Oncology, St Jude Children's Research Hospital, Memphis, TN

## Abstract

**PURPOSE:**

To characterize the association between neurocognitive outcomes (memory and processing speed) and radiation (RT) dose to the hippocampus, corpus callosum (CC), and frontal white matter (WM) in children with medulloblastoma treated on a prospective study, SJMB03.

**PATIENTS AND METHODS:**

Patients age 3-21 years with medulloblastoma were treated at a single institution on a phase III study. The craniospinal RT dose was 23.4 Gy for average-risk patients and 36-39.6 Gy for high-risk patients. The boost dose was 55.8 Gy to the tumor bed. Patients underwent cognitive testing at baseline and once yearly for 5 years. Performance on tests of memory (associative memory and working memory) and processing speed (composite processing speed and perceptual speed) was analyzed. Mixed-effects models were used to estimate longitudinal trends in neurocognitive outcomes. Reliable change index and logistic regression were used to define clinically meaningful neurocognitive decline and identify variables associated with decline.

**RESULTS:**

One hundred and twenty-four patients were eligible for inclusion, with a median neurocognitive follow-up of 5 years. Mean right and left hippocampal doses were significantly associated with decline in associative memory in patients without posterior fossa syndrome (all *P* < .05). Mean CC and frontal WM doses were significantly associated with decline in both measures of processing speed (all *P* < .05). Median brain substructure dose–volume histograms were shifted to the right for patients with a decline in associative memory or processing speed. The odds of decline in associative memory and composite processing speed increased by 23%-26% and by 10%-15% for every 1-Gy increase in mean hippocampal dose and mean CC or frontal WM dose, respectively.

**CONCLUSION:**

Increasing RT dose to the CC or frontal WM and hippocampus is associated with worse performance on tests of processing speed and associative memory, respectively. Brain substructure–informed RT planning may mitigate neurocognitive impairment.

## INTRODUCTION

Medulloblastoma survivors are at risk for cognitive impairment secondary to treatment-related and disease-related factors. Treatment entails maximal safe resection, craniospinal irradiation (CSI) with a boost to the primary tumor bed, and chemotherapy.^1^ Higher CSI doses and younger age at treatment are associated with poorer cognitive outcomes.^2^ The effects of neurocognitive impairment are pervasive, affecting academic performance,^3^ social-emotional functioning,^4^ and the ability to live independently as an adult.^5^

**CONTEXT**

**Key Objective**
Medulloblastoma survivors are at risk for cognitive impairment secondary to radiation (RT). Brain substructures such as the hippocampus, corpus callosum (CC), and frontal white matter (WM) are particularly vulnerable to RT injury and are associated with specific neurocognitive functions. Using data from SJMB03, a phase III study in which children with histologically confirmed medulloblastoma received craniospinal RT, we investigated the relation between the following: (1) hippocampal dose and memory, (2) CC dose and processing speed, and (3) frontal WM dose and processing speed.
**Knowledge Generated**
Higher mean dose to the hippocampi and CC or frontal WM was associated with worse performance on tests of associative memory and processing speed, respectively.
**Relevance**
This study sets the stage for implementing substructure-informed RT planning in future medulloblastoma protocols with the goal of reducing deficits in memory and processing speed, ultimately improving the quality of life of medulloblastoma survivors.

The hippocampus and white matter (WM) are particularly vulnerable to radiation (RT) injury. The hippocampus plays a primary role in encoding new memories,^6-8^ and neurogenesis within the hippocampus is impaired after cranial irradiation.^9-11^ Hippocampal avoidance in adults undergoing whole-brain RT has resulted in better memory preservation when compared with standard of care.^12^ In children with low-grade brain tumors, increased hippocampal dose is associated with worse memory performance.^13,14^ With respect to WM, there is a correlation between decreased WM integrity and RT dose, with the largest changes occurring in the corpus callosum (CC) and frontal WM.^15-17^ Decreased WM integrity within these regions correlates with specific neurocognitive domains, such as attention and processing speed.^17,18^ Overall, these data suggest that the hippocampus, CC, and frontal WM are particularly vulnerable to injury from RT and are associated with specific neurocognitive functions.

The increased conformality of modern RT techniques and the reduction in medulloblastoma boost volume from whole posterior fossa to tumor bed provide an opportunity to minimize RT injury to brain substructures critical for neurocognitive function.^19^ A better understanding of the relation between dose to brain substructures and cognitive outcomes in children will result in RT plans that are optimized to preserve neurocognition. The primary aim of this study was to use data from SJMB03 (ClinicalTrials.gov identifier: NCT00085202), a phase III study in which children with histologically confirmed medulloblastoma received CSI with a focal boost to the tumor bed, to investigate the relation between the following: (1) hippocampal dose and memory, (2) CC dose and processing speed, and (3) frontal WM dose and processing speed. We hypothesized that increasing dose to the hippocampus and CC or frontal WM would adversely affect memory and processing speed, respectively. The secondary aim was to understand how RT affected the growth of these substructures by comparing volumetric change in patients with that in healthy participants.

## PATIENTS AND METHODS

### Study Population

#### SJMB03 patients.

**FIG A1.**
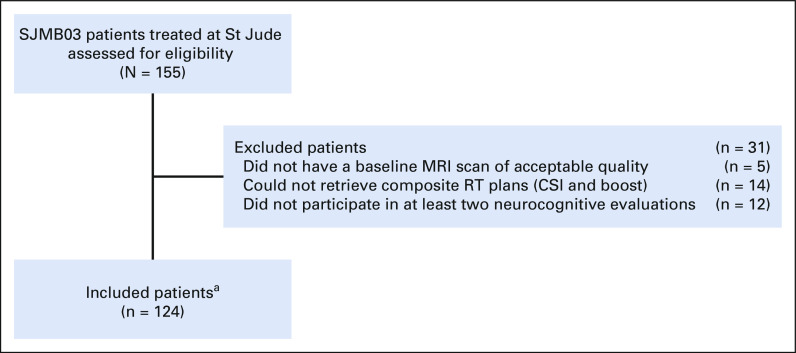
Flow diagram. ^a^There were no statistically significant differences in age, sex, and risk group when comparing SJMB03 patients excluded from this study with those included in this study (age, *P* = .107; sex, *P* = .942; risk group, *P* = .872). CSI, craniospinal irradiation; MRI, magnetic resonance imaging; RT, radiation.

Eligible patients included children with medulloblastoma who were age 3-21 years at diagnosis and were enrolled on SJMB03 at St Jude Children's Research Hospital (St Jude; N = 155; Appendix Fig A1, online only). Patients were enrolled at St Jude from September 2003 to June 2013. Patients enrolled and treated at non-St Jude sites were not included in this study. SJMB03 trial schema and eligibility criteria have been reported previously.^20^ In brief, patients with average-risk disease (M0 and gross total volume or near total volume) received 23.4 Gy of CSI and those with high-risk disease (M+ or subtotal volume) received 36-39.6 Gy of CSI. All patients received photon therapy, with a 55.8 Gy boost to the tumor bed, using a 1-cm clinical target volume margin. Magnetic resonance imaging (MRI; T1, T2, and/or fluid-attenuated inversion recovery) was used to define the boost target. Metastases > 0.5 cm received a boost of 50.4-54 Gy. One high-risk patient was treated to 41.4 Gy CSI because of extensive metastases. All patients received adjuvant chemotherapy, scheduled 6 weeks after completion of RT and consisting of four cycles of high-dose cyclophosphamide, cisplatin, and vincristine with stem-cell support. The following clinical variables were extracted from the medical record database: age at study enrollment, sex, number of surgeries, completion of adjuvant chemotherapy, presence of hydrocephalus (defined as requiring a shunt, external ventricular drain, or endoscopic third ventriculostomy), hearing loss (defined as > 25 dB of hearing loss at 2,000 Hz), posterior fossa syndrome (PFS; defined according to the 2016 Iceland Delphi Consensus Conference),^21^ median household income on the basis of residential zip code (estimated using 2006-2010 data from the American Community Survey^22^), and molecular subgroup (determined by DNA methylation–based classification^23^).

Patients were excluded if the cumulative RT plan (CSI and boost) could not be retrieved (n = 14); if the baseline pre-RT MRI scan was of poor quality, preventing delineation of brain substructures (n = 5); or if the patient had completed fewer than two neurocognitive evaluations (n = 12; Appendix Fig A1).

SJMB03 was approved by the St Jude Institutional Review Board. Written informed consent was obtained from patients, parents, or guardians.

#### Healthy participants.

Eligible participants were age 6-25 years and had no major psychiatric, neurologic, or medical diagnoses. Participants were enrolled from October 16, 2007, to June 08, 2010, with a sex bias to match the patient population, and were recruited from the community. They were scheduled for three yearly noncontrast MRI scans (years 1, 2, and 3) without anesthesia. Written informed consent was obtained from participants, parents, or guardians. The imaging Protocol (online only) was approved by the St Jude Institutional Review Board.

### Dose and Volume Analysis

Dose–volume histogram (DVH) data were extracted from cumulative RT plans. Each substructure was contoured on the postoperative pre-RT MRI, and the MRI was fused to the simulation CT. Contours were primarily based on three-dimensional (3D) T1 postcontrast MRI sequences. The hippocampus and CC were contoured according to the RTOG 0933 atlas and published guidelines,^24^ respectively, by a board-certified RT oncologist. Frontal WM was autosegmented using FreeSurfer Software^25^ (Laboratory for Computational Neuroimaging at the Athinoula A. Martinos Center for Biomedical Imaging; Boston, MA) and verified manually.

For volumetric analysis of the SJMB03 population, the 2-year follow-up 3D T1 postcontrast MRI sequence was used for contouring if the image quality was adequate (n = 101). For volumetric analysis of healthy participants, 3D T1 noncontrast MRI sequences were used for contouring if image quality was adequate and both years 1 and 2 or years 1 and 3 scans were available (n = 82). The slice thickness was ≤ 1 mm for all MRI scans.

### Neurocognitive Assessment

Patients underwent neurocognitive testing at baseline, defined as after surgical resection and before completion of RT, and once yearly for 5 years. The testing battery was consistent across all time points, and the results from the full battery have been reported previously.^2,26^ For this study, specific measures from the Woodcock–Johnson Tests of Cognitive Abilities, Third Edition,^27^ were chosen to characterize memory and processing speed. For memory, these measures included Visual Auditory Learning (associative memory) and Numbers Reversed (working memory). For processing speed, these measures included Visual Matching (perceptual speed) and a composite measure of processing speed that combined Visual Matching and Decision Speed (semantic processing speed). Age-adjusted standard scores on the basis of a large, representative normative sample have a population mean of 100 and a standard deviation (SD) of 15. Lower scores indicate worse performance. Patients with progressive disease discontinued protocol-based neurocognitive testing.

### Statistical Analysis

#### Association between cognitive function, brain substructure dose, and clinical variables.

The outcome variable was neurocognitive performance, as described above. Follow-up time was calculated from RT start to the last neurocognitive test. Neurocognitive evaluations completed after disease progression were excluded from the analysis. Linear mixed-effects models were used to estimate longitudinal trends in these neurocognitive outcomes over time. Each patient was treated as a cluster, and the intercept was assumed to be random among patients. First, a series of univariable linear mixed-effects models were built for each covariate separately. These models included covariates and covariate-by-time interaction terms (slope). Second, clinical covariates and their interactions with time were included in the multivariable model if their interactions with time were significant on the first step (*P* < .05); however, age was always included in the multivariable model. Additionally, if the interaction of a dosimetric covariate with time was significant on univariate analysis (*P* < .05), it was carried forward to the multivariable model. Separate multivariable models were created for the right and left hippocampus, the right and left frontal WM, and the subdivisions of the CC (genu, body, and splenium). For each substructure, the Akaike information criterion (AIC) and Bayesian information criterion (BIC) were used to compare multivariable models with different dosimetric parameters (ie, the mean dose [D_mean_] and the volumes receiving 30, 35, 40, and 45 Gy), with lower AIC and BIC values indicating better models. The multivariable model with D_mean_ resulted in the lowest AIC and BIC for each substructure, and a final stepwise selection was conducted to remove covariate(s) that turned insignificant in the multivariable model.

#### Association between substructure D_mean_ and clinically meaningful neurocognitive decline.

Because many factors can affect neurocognitive scores on repetitive testing, the reliable change index (RCI) was used to identify patients with a clinically meaningful decline from baseline for each neurocognitive measure.^28,29^ RCI was calculated as follows:

$$\text{RCI}=\;\frac{{\text{T}}_{1\;}-{\text{T}}_{2}}{\text{SED}}\text{,}$$

where SED is the SE of difference calculated from the SD of the test and the test reliability coefficient,^27^ T_1_ is the baseline score, and T_2_ is the score at last neurocognitive follow-up. RCI results in a *z*-score similar to the SD index, and any score below –1.645 signifies a clinically meaningful decline.^29^ Logistic regression was used to identify mean substructure doses associated with clinically meaningful declines. Only patients with both baseline and follow-up measurements were included in this analysis.

#### Comparing DVHs of patients with and without clinically meaningful neurocognitive decline.

Permutation testing was performed to determine whether the median population DVH of patients with clinically meaningful neurocognitive decline was significantly different from that of patients without decline with an α = .05 significance level (ie, ${\text{H}}_{0}:\tilde{{\text{D}}_{\text{Decline}}}=\tilde{{\text{D}}_{\text{No decline}}};\;{\text{H}}_{1}:\;\tilde{{\text{D}}_{\text{Decline}}}\neq \tilde{{\text{D}}_{\text{No decline}}}$). Permutation testing enables multiple comparisons without the need for additional correction and provides a nonparametric method to establish statistical significance.^30-32^ Our permutation test compared the test statistic, T,

$$\text{T}=\overset{{\text{D}}_{\text{max}}}{\underset{{\text{D}}_{\text{i}}=0}{\sum }}\left(\tilde{{\text{V}}_{\text{Decline}}}\left({\text{D}}_{\text{i}}\right)-\tilde{{\text{V}}_{\text{No decline}}}\left({\text{D}}_{\text{i}}\right)\right)\text{,}$$

where D_max_ is the maximum dose magnitude in the DVH and $\tilde{{\text{V}}_{\text{Decline}}}\left({\text{D}}_{\text{i}}\right)$ $\tilde{{\text{V}}_{\text{No decline}}}\left({\text{D}}_{\text{i}}\right)$ are the median cumulative volume magnitudes at D_i_ for the decline and no decline cohorts of each permutation, respectively.

#### Comparing change in brain substructure volumes of patients and healthy participants.

The outcome variable was the annual volumetric change in each brain substructure. For patients, volumetric data were extracted from the baseline MRI (acquired after surgery and before RT) and from the 2-year follow-up MRI. For control participants, volumetric data were extracted from two MRI scans scheduled 1-2 years apart and the baseline MRI was defined as the initial MRI scan. Linear regression was used to model the annual volumetric change within each treatment group (patients *v* healthy participants), adjusting for baseline volume, age, and sex. Baseline volume was included as a covariate because it differed significantly between patients and healthy participants. All statistical analyses were performed using R version 4.0.2.

## RESULTS

### Patient Characteristics

**FIG 1.**
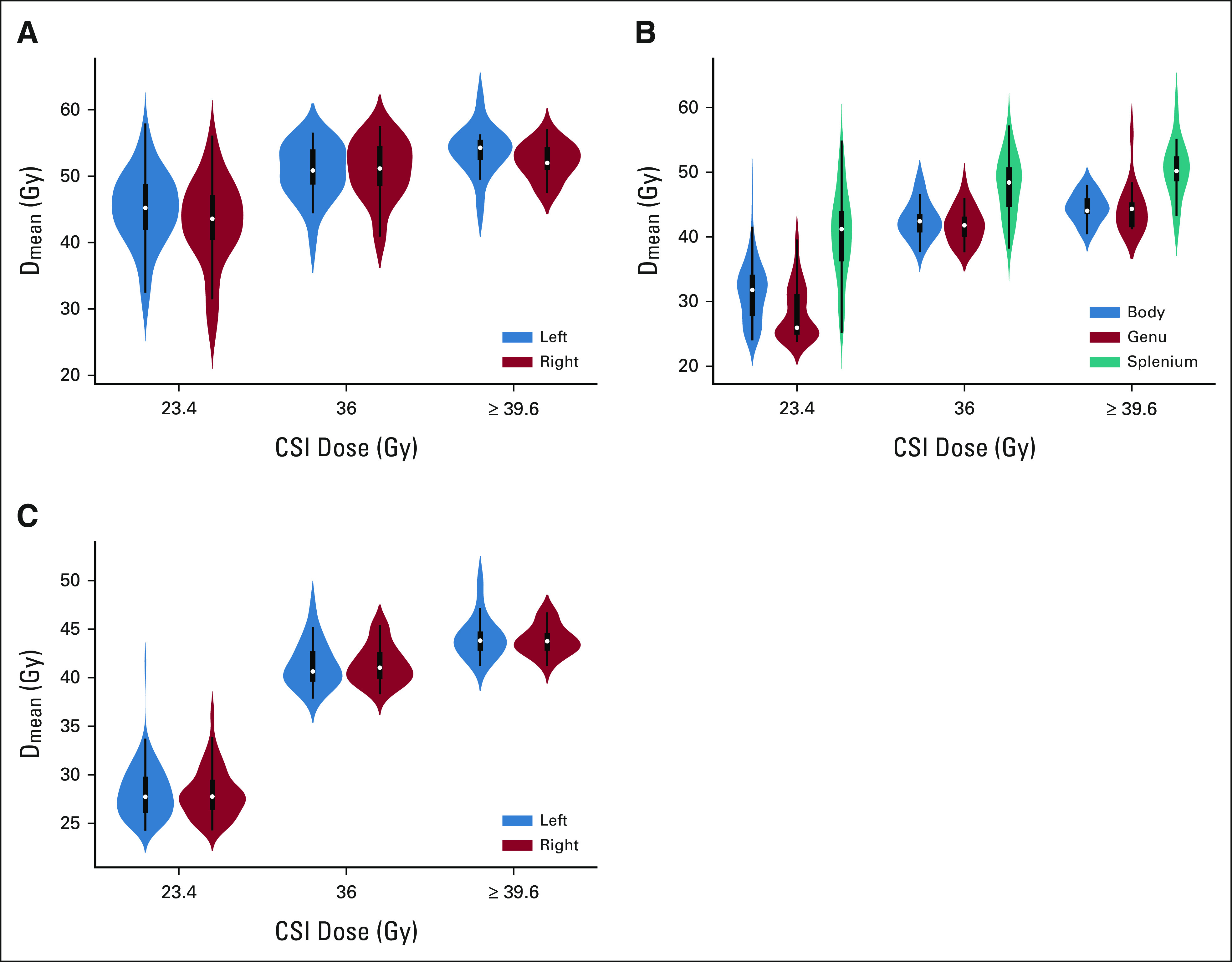
Violin plots of D_mean_ to (A) the hippocampus, (B) the CC, and (C) the frontal WM, stratified by craniospinal dose. CC, corpus callosum; CSI, craniospinal irradiation; D_mean_, mean dose; WM, white matter.

**FIG A2.**
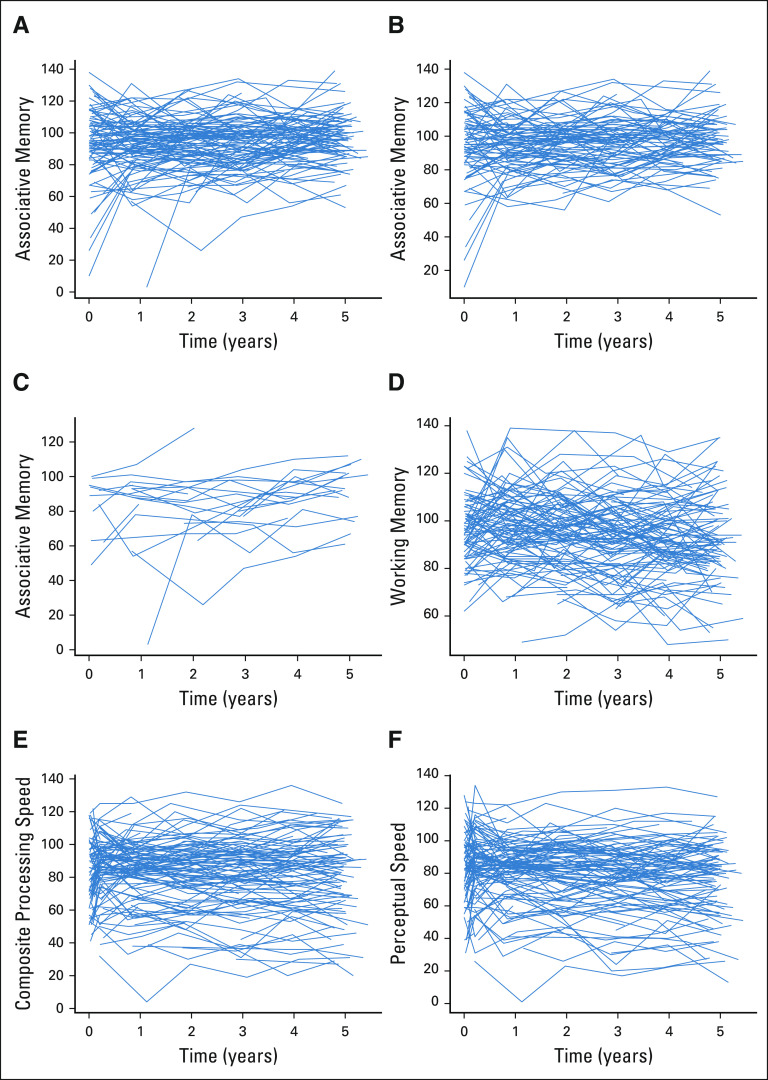
Raw individual patient data for (A) associative memory, (B) associative memory for patients without PFS, (C) associative memory for patients with PFS, (D) working memory, (E) composite processing speed, and (F) perceptual speed. PFS, posterior fossa syndrome.

A total of 124 patients were eligible for inclusion in the study. Descriptive statistics are presented in Table 1. The median neurocognitive follow-up was 5 years. The number of patients with neurocognitive testing at each time point and individual patient data are shown in Appendix Table A1 and Figure A2 (online only), respectively. Eighty-four patients (68%) were treated on the average-risk arm and received 23.4 Gy CSI. The remainder were treated to ≥ 36 Gy CSI with or without intracranial metastatic boosts. Substructure D_mean_ varied greatly among patients, even within the 23.4 Gy CSI group (Fig 1). Patients treated with ≥ 36 Gy CSI had higher D_means_ across all substructures compared with those treated with 23.4 Gy CSI (Appendix Table A2, online only). Within average-risk patients, sonic hedgehog subgroup (n = 15) was associated with lower right hippocampal D_mean_ compared with nonsonic hedgehog subgroups (n = 62; 34.9 Gy *v* 45.3 Gy, *P* = .002).

**TABLE 1.**
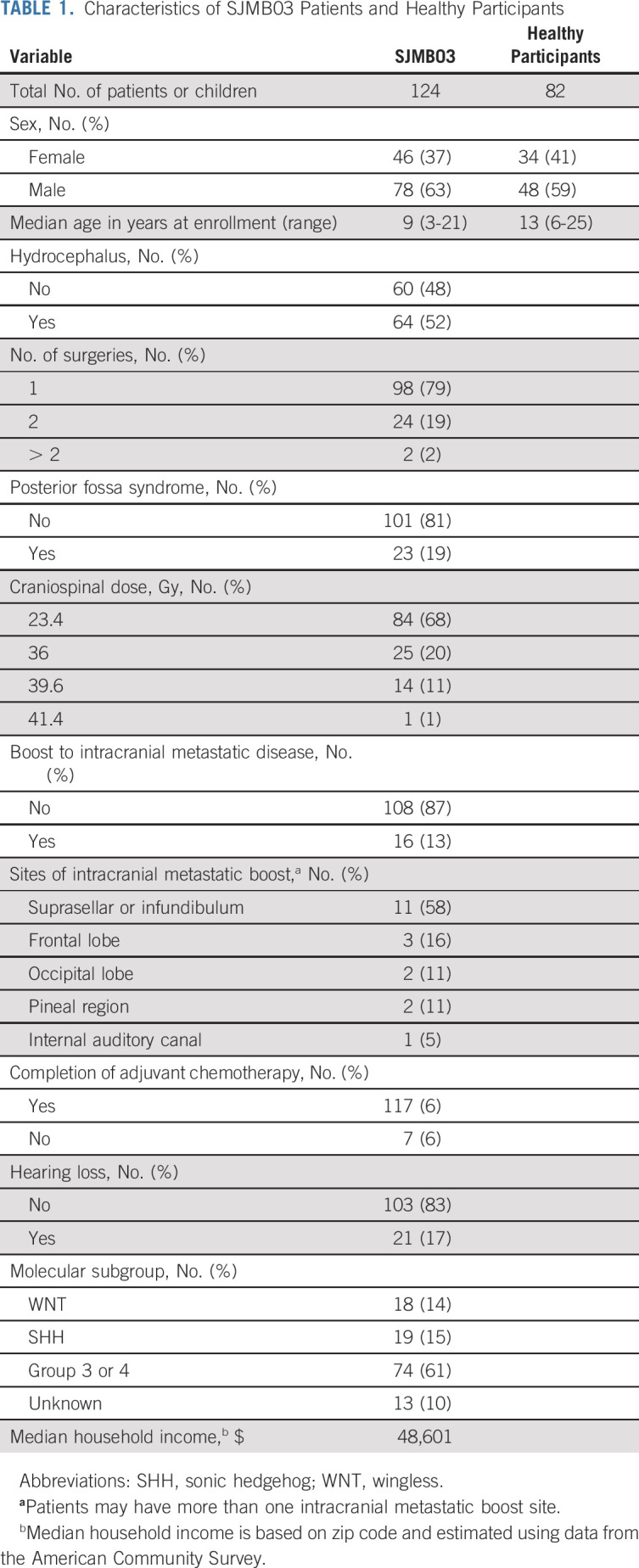
Characteristics of SJMB03 Patients and Healthy Participants

### Hippocampus and Memory

**FIG 2.**
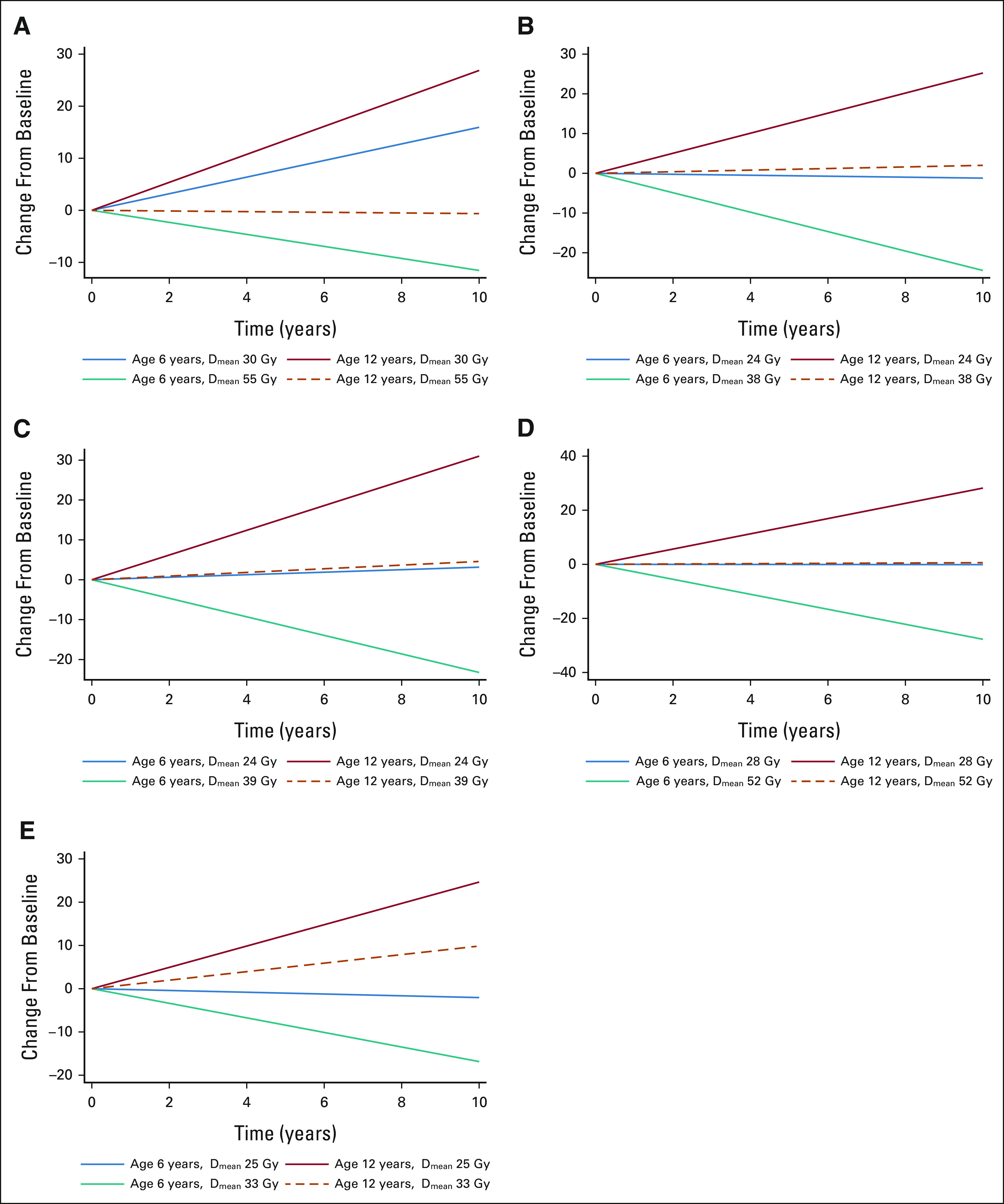
Association between age, brain substructure, and neurocognitive outcomes for (A) the right hippocampus and associative memory, (B) the CC genu and composite processing speed, (C) the CC body and composite processing speed, (D) the CC splenium and composite processing speed, and (E) the right frontal WM and composite processing speed. Data from the left hippocampus and left frontal WM are not shown because the results are similar to those for the contralateral side. The doses displayed represent the fifth and 95th percentiles of the substructure D_mean_ distribution in average-risk patients. CC, corpus callosum; D_mean_, mean dose; WM, white matter.

Appendix Tables A3-A5 (online only) list univariable models for associative memory and working memory. Because PFS was significantly associated with improving associative memory performance on univariable analysis, we analyzed multivariable models of patients with and without PFS separately. In patients without PFS (n = 99), hippocampal D_mean_ was significantly associated with decline in associative memory, after accounting for other significant variables such as age and sex (Table 2). In patients with PFS (n = 19), no clinical or dosimetric variables were associated with the longitudinal trend of associative memory. With respect to working memory, only age and sex had significant time interactions on multivariable analysis (Table 2). Figure 2A demonstrates how associative memory performance changes with hippocampal D_mean_, age, and time in patients without PFS.

**TABLE 2.**
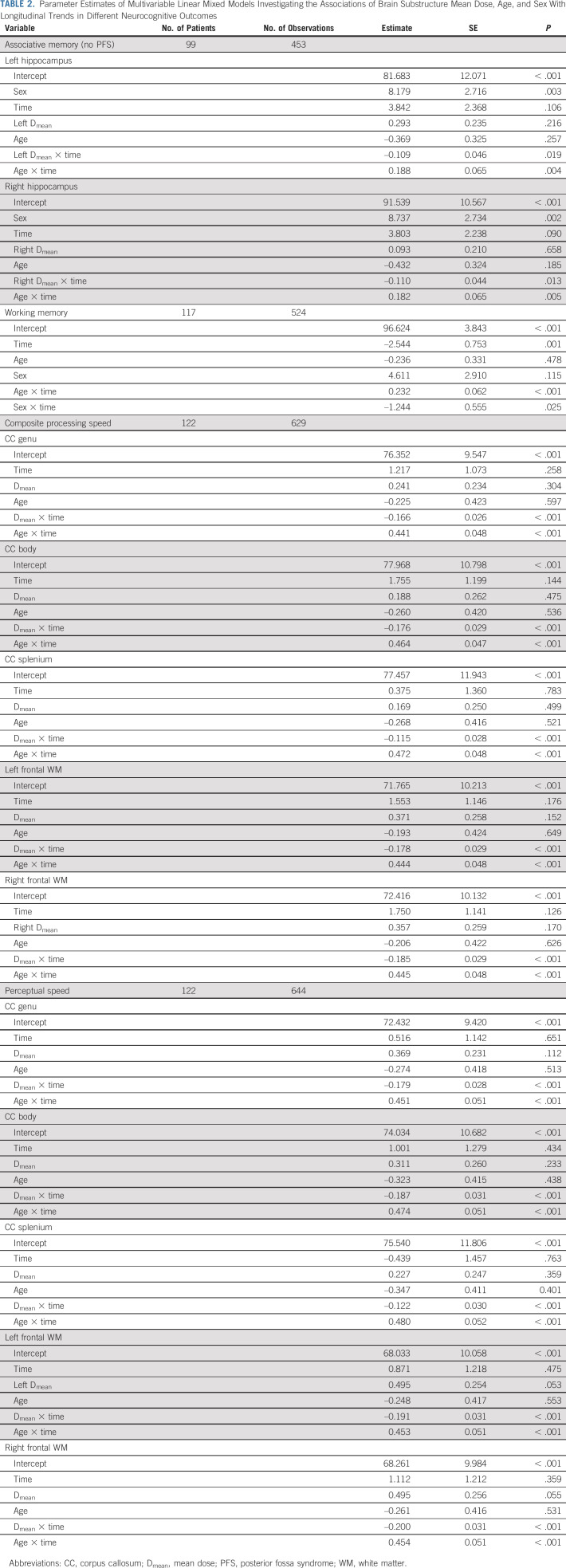
Parameter Estimates of Multivariable Linear Mixed Models Investigating the Associations of Brain Substructure Mean Dose, Age, and Sex With Longitudinal Trends in Different Neurocognitive Outcomes

### CC, Frontal WM, and Processing Speed

CC (genu, body, and splenium) and frontal WM D_means_ were associated with declining scores on tests of composite processing and perceptual speed, after accounting for age (Table 2 and Figs 2B-2E). Although hearing loss and hydrocephalus were also associated with declining composite processing and perceptual speed on univariable analysis (Appendix Tables A6 and A7, online only), the associations were not significant on multivariable analysis.

### Brain Substructure DVH and Neurocognitive Decline

**FIG 3.**
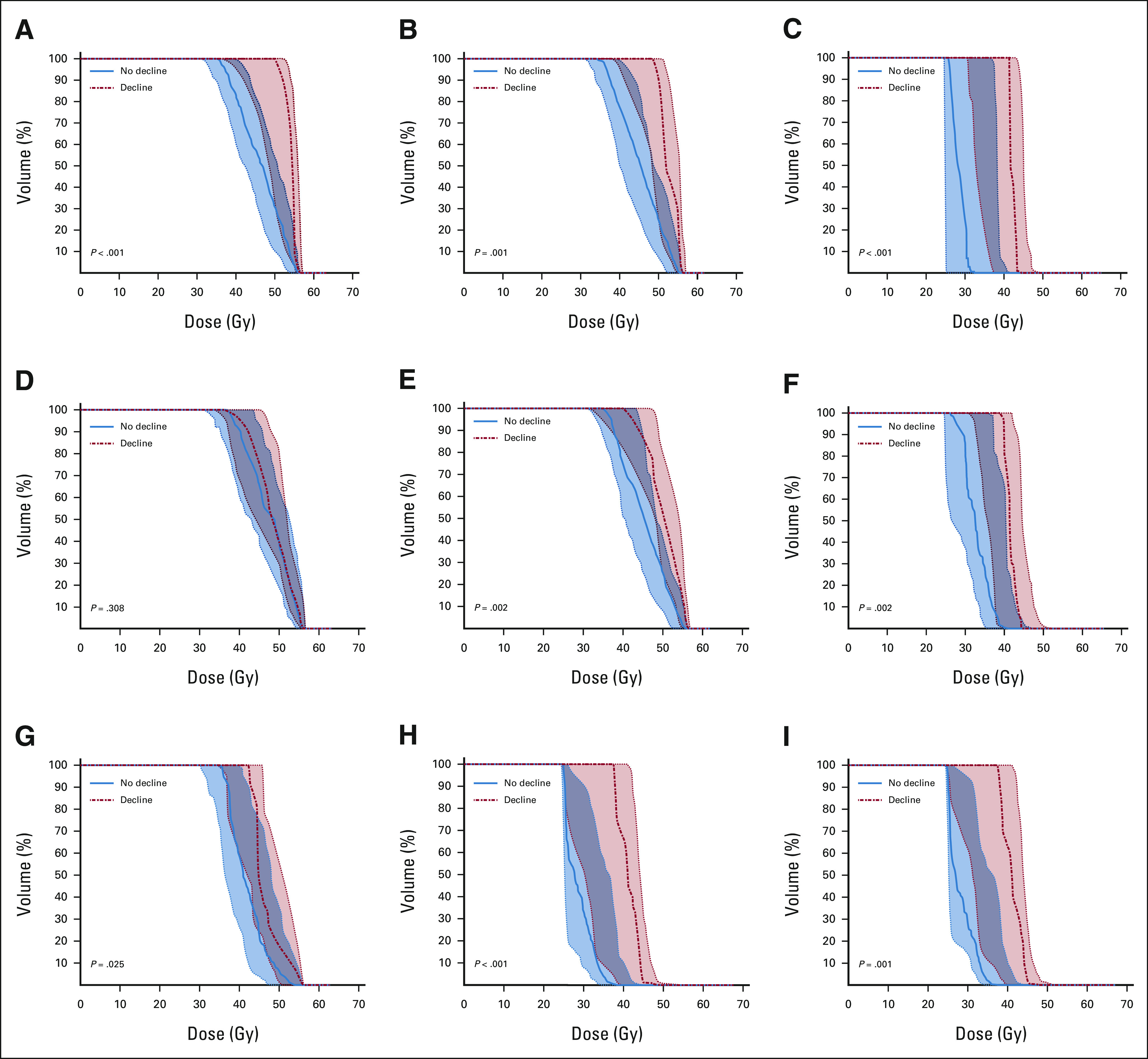
Median population DVHs for patients exhibiting no decline (solid line, blue) compared with patients exhibiting decline (dotted line, red) for the following brain substructures and neurocognitive outcomes: (A) the left hippocampus and associative memory, (B) the right hippocampus and associative memory, (C) the CC genu and composite processing speed, (D) the left hippocampus and working memory, (E) the right hippocampus and working memory, (F) the CC body and composite processing speed, (G) the CC splenium and composite processing speed, (H) the right frontal WM and composite processing speed, and (I) the left frontal WM and composite processing speed. The shaded region represents the 25th-75th percentile. CC, corpus callosum; DVH, dose–volume histogram; WM, white matter.

**FIG 4.**
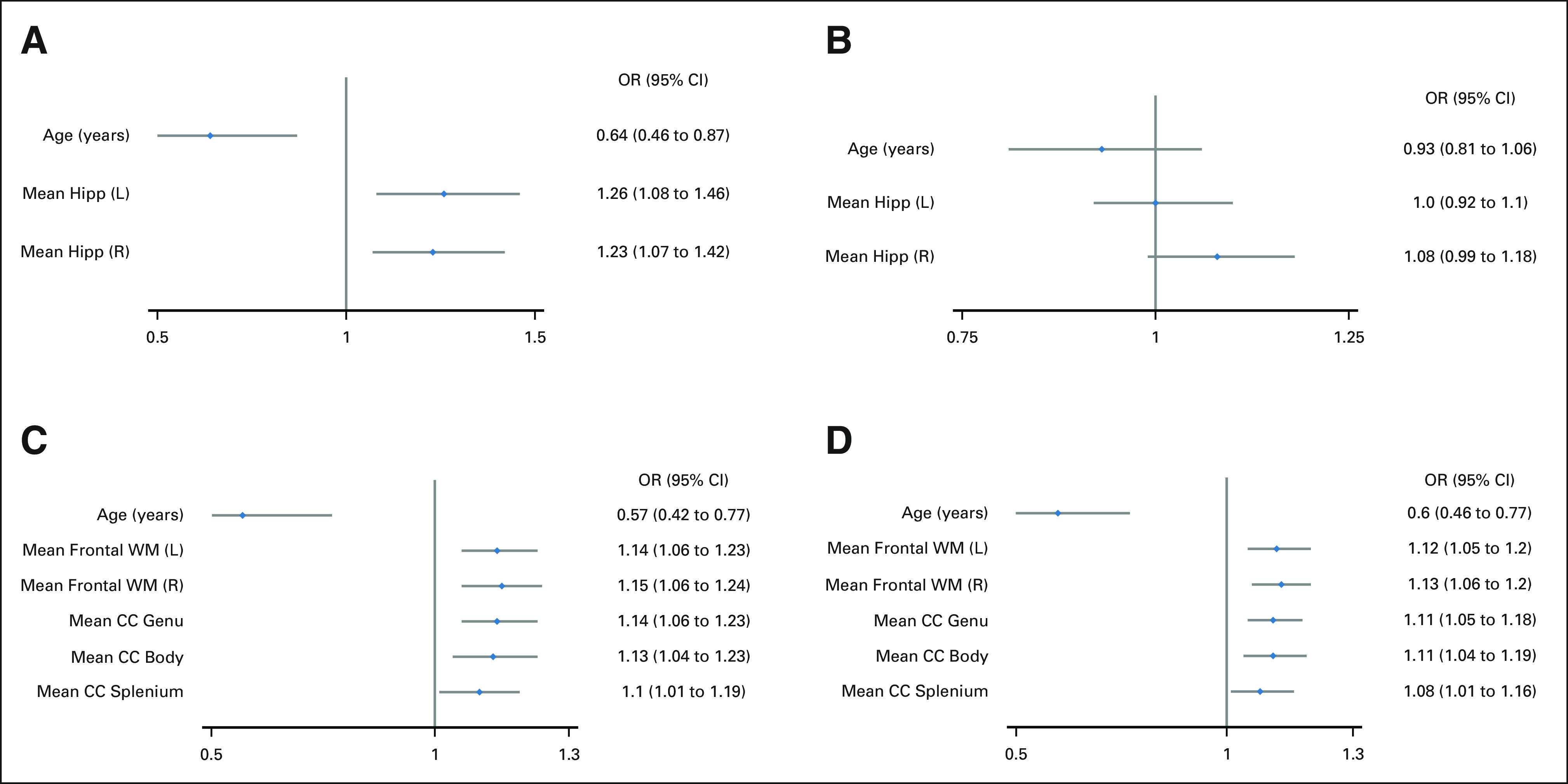
The odds of experiencing neurocognitive decline on the basis of age and D_mean_ with respect to (A) associative memory, (B) working memory, (C) composite processing speed, and (D) perceptual speed. All variables are continuous, and mean doses are represented in Gy. CC, corpus callosum; D_mean_, mean dose; Hipp, hippocampus; L, left; OR, odds ratio; R, right; WM, white matter.

**FIG A3.**
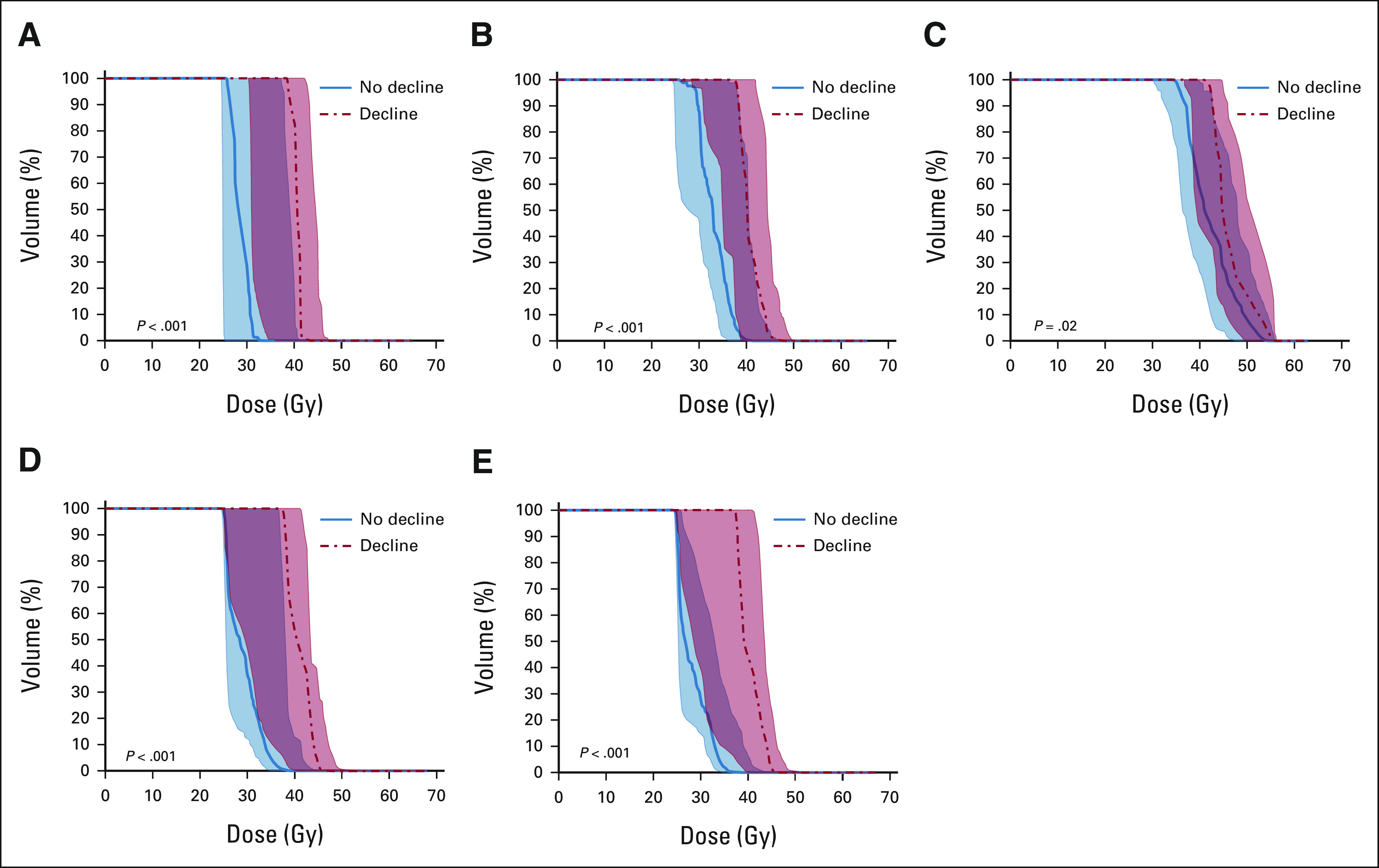
Median population DVHs for patients exhibiting no decline (solid line, blue) compared with patients exhibiting decline (dotted line, red) in perceptual speed for the following brain substructures: (A) the CC genu, (B) the CC body, (C) the CC splenium, (D) the left frontal WM, and (E) the right frontal WM. The shaded region represents the 25th-75th percentile. CC, corpus callosum; DVH, dose–volume histogram; WM, white matter.

The number of patients experiencing meaningful declines in associative memory, working memory, composite processing speed, and perceptual speed were 11 (13%), 18 (22%), 17 (18%), and 24 (24%), respectively (Appendix Table A8, online only). Figure 3 demonstrates population DVHs of patients with and without meaningful decline along with the corresponding *P* values of the permutation tests. The hippocampus median DVH was shifted to the right for patients with a decline in associative memory (left hippocampus: *P* < .001; right hippocampus *P* = .001; Figs 3A and 3B). However, with respect to working memory, the left hippocampus DVH did not differ between patients with and without decline (*P* = .308; Fig 3D), but the right hippocampus DVH did (*P* = .002; Fig 3E). The CC and frontal WM median DVHs were also shifted to the right for patients with a decline in composite processing speed and perceptual speed (all *P* ≤ .025; Figs 3C and 3F-3I, and Appendix Fig A3, online only). The odds of decline in associative memory increased by 23%-26% for every 1-Gy increase in hippocampal D_mean_ (Fig 4A). The odds of a decline in composite processing speed and perceptual speed increased by 10%-15% and 8%-12%, respectively, for every 1-Gy increase in CC or frontal WM D_mean_ (Figs 4C and 4D).

### Substructure Volumetric Analysis of Patients Versus Healthy Participants

**FIG A4.**
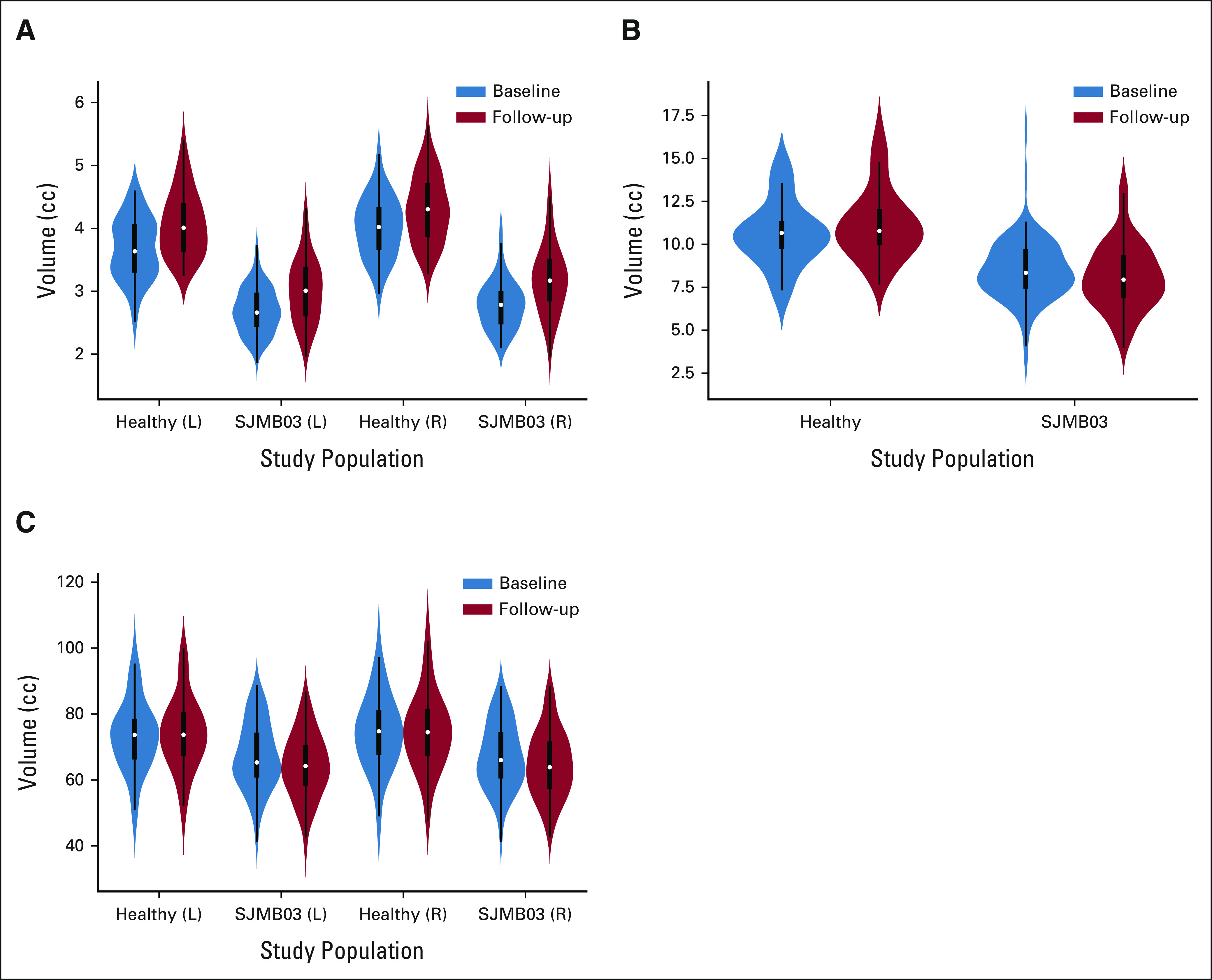
Violin plots comparing patient (SJMB03) and healthy participant (healthy) substructure volumes for (A) the hippocampus, (B) the CC, and (C) the frontal WM. The follow-up time was 1-2 years after the baseline time point. CC, corpus callosum; L, left; R, right; WM, white matter.

Participant characteristics are listed in Table 1. Appendix Figure A4 (online only) shows baseline and follow-up volumes, acquired 1-2 years from baseline. After accounting for baseline volume, age, and sex, patients demonstrated decreased annual growth in the first 2 years after therapy across all brain substructures when compared with healthy participants (Appendix Table A9, online only).

## DISCUSSION

Delivering RT without disrupting cognitive function represents a central dilemma in neuro-oncology. Historically, strategies to mitigate neurocognitive impairment have included reducing the craniospinal dose, reducing the boost volume, and using conformal RT to reduce the dose to normal brain.^20,33,34^ There is increasing preclinical and clinical evidence that brain substructures such as the hippocampus, CC, and frontal WM are differentially sensitive to RT injury.^24,35-37^ To investigate the relation between brain substructure doses and specific neurocognitive outcomes, we used data from SJMB03. We made four important discoveries: (1) mean substructure dose varied greatly among patients, even within average-risk patients; (2) D_mean_ to certain brain substructures was correlated with longitudinal change of specific neurocognitive functions; (3) substructure DVHs were shifted to the right for patients who experienced clinically meaningful decline, as compared with those who did not; and (4) brain substructure growth was impaired in patients, as compared with healthy participants. Patients also had reduced baseline substructure volumes, potentially explained by hydrocephalus.^38,39^ Overall, these data highlight the possibility of cognitively optimizing RT plans, shifting the RT planning paradigm from one that is substructure-naïve to the one that is substructure-informed. This work is particularly timely because hippocampal dose reduction is feasible in the era of proton therapy and tumor bed boosts if the hippocampus is contoured as an organ at risk and proton beam angles are carefully chosen to minimize both hippocampal irradiation and beam path length. Coupling such technological advances with substructure-informed RT planning may provide the best neurocognitive outcomes.

Declining associative memory performance correlated with increasing hippocampal dose for patients without PFS. The odds of a meaningful decline in associative memory increased by 23%-26% for every 1-Gy increase in hippocampal D_mean_. For patients with PFS, associative memory performance did not correlate with hippocampal dose and the presence of PFS was associated with improvement in scores over time. This improvement probably reflects the timeline of PFS recovery. The severity of symptoms such as mutism, ataxia, and emotional lability^40^ is greatest in the immediate postoperative period; however, symptoms may improve in subsequent months.^41^ Unlike associative memory, working memory did not correlate with hippocampal dose after accounting for age and sex. Working memory is highly dependent on the prefrontal cortex,^42^ in addition to the hippocampus, and entails short-term active manipulation of information. Associative memory requires learning and recall of information and is more dependent on encoding supported by the hippocampus.

Declining processing speed performance correlated with increasing dose to the CC and frontal WM. The odds of a meaningful decline in composite processing speed and perceptual speed increased by 10%-15% and 8%-12%, respectively, for every 1-Gy increase in CC or frontal WM D_mean_. Reducing dose to these regions will be difficult without considering new targeting approaches that carve out deep frontal WM from the craniospinal target or reduce the craniospinal dose, as is being tested in patients with nonmetastatic wingless medulloblastoma (ClinicalTrials.gov identifier: NCT01878617, NCT02724579, and NCT04474964). Results from ACNS0331 suggest that novel strategies of dose reduction should be cautiously applied in selected patients on the basis of their molecular genomic risk profile.^43^

This was a retrospective study of prospectively collected data with limitations inherent to the study design (eg, data on handedness were not available). We did not account for practice effects; however, measures used had acceptable test–retest reliability for yearly testing intervals. As doses to different brain regions correlate with one another, we pursued a knowledge-based, hypothesis-driven approach rather than a data-mining approach, limiting our testing to substructures for which there were scientific data correlating the anatomic structure with a neurocognitive function. Finally, intracranial disease burden, independent of RT, may affect neurocognitive outcomes; however, gross disease was either resected or boosted with RT.

This study sets the stage for implementing substructure-informed RT planning in future medulloblastoma protocols. However, more work is needed to understand how RT alters dynamic processes in a child's brain, including the establishment of new neural connections, synaptic pruning, and progressive myelination. Synergizing biologic and clinical understanding of these processes with technological advancements in RT will allow us to deliver the most efficacious therapy with the least amount of damage, improving the lives of medulloblastoma survivors.

## Figures and Tables

**TABLE A1.**
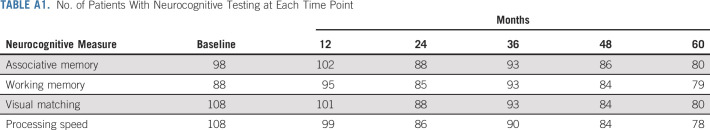
No. of Patients With Neurocognitive Testing at Each Time Point

**TABLE A2.**
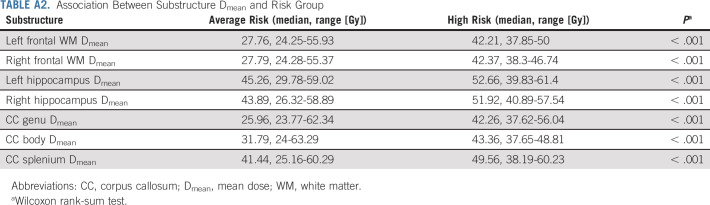
Association Between Substructure D_mean_ and Risk Group

**TABLE A3.**
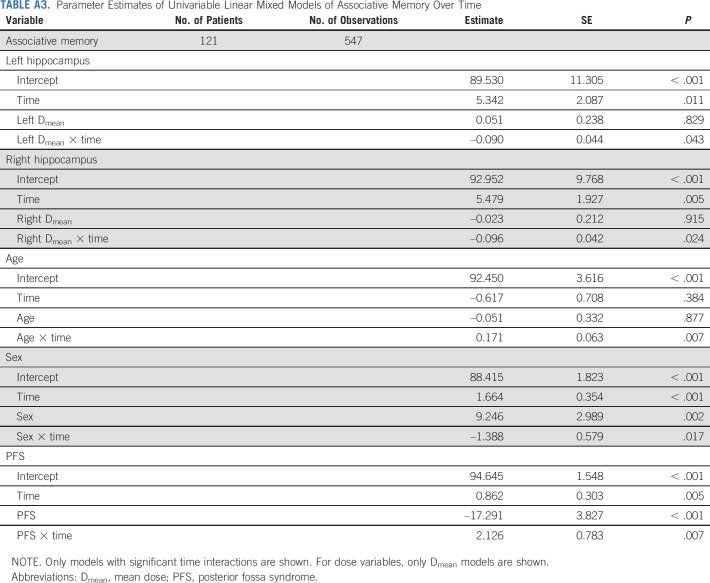
Parameter Estimates of Univariable Linear Mixed Models of Associative Memory Over Time

**TABLE A4.**
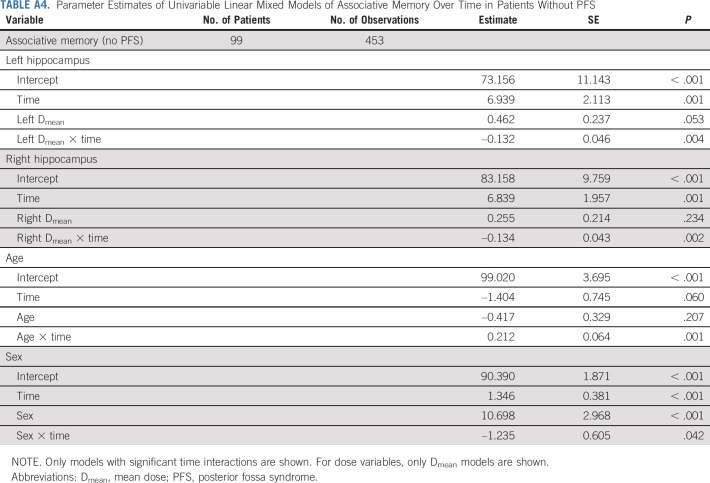
Parameter Estimates of Univariable Linear Mixed Models of Associative Memory Over Time in Patients Without PFS

**TABLE A5.**
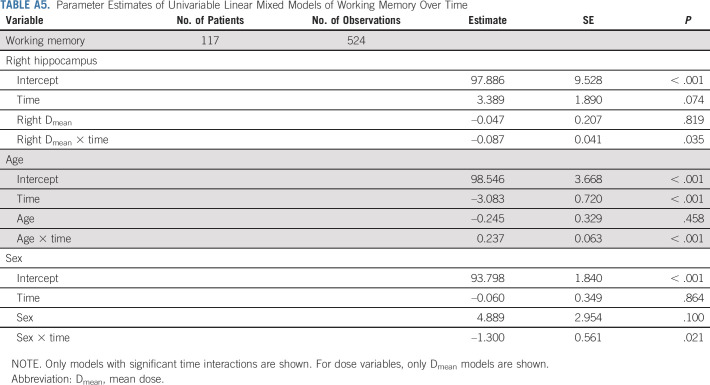
Parameter Estimates of Univariable Linear Mixed Models of Working Memory Over Time

**TABLE A6.**
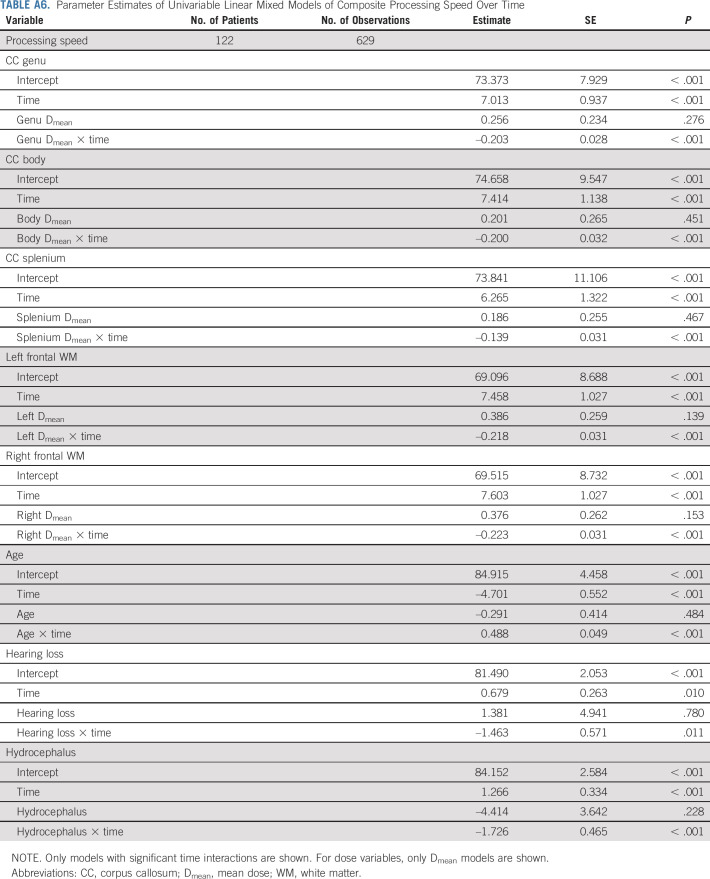
Parameter Estimates of Univariable Linear Mixed Models of Composite Processing Speed Over Time

**TABLE A7.**
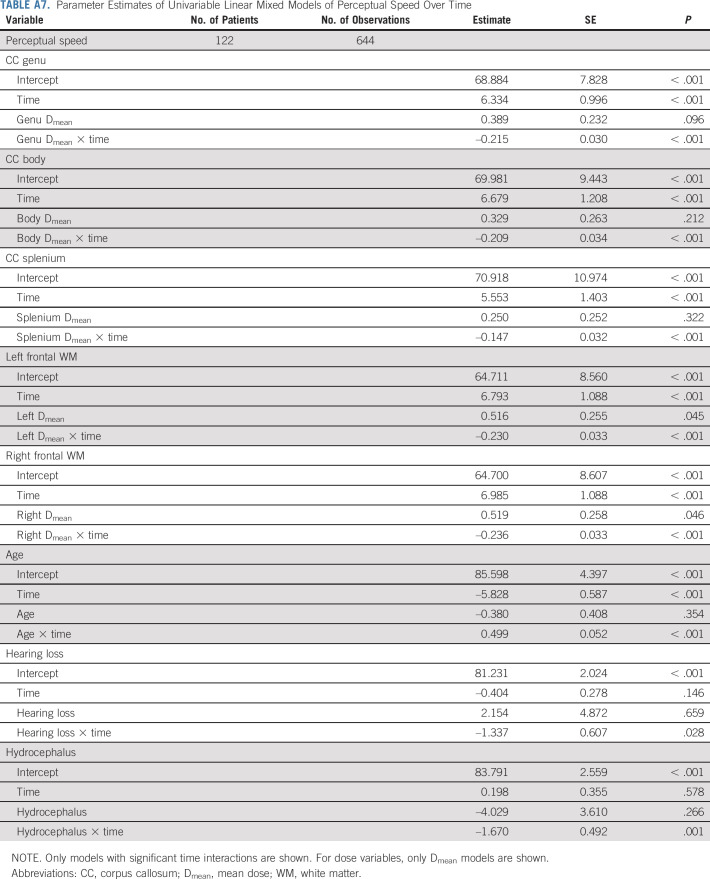
Parameter Estimates of Univariable Linear Mixed Models of Perceptual Speed Over Time

**TABLE A8.**
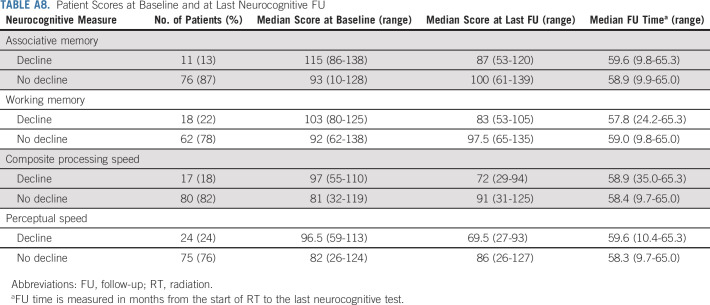
Patient Scores at Baseline and at Last Neurocognitive FU

**TABLE A9.**
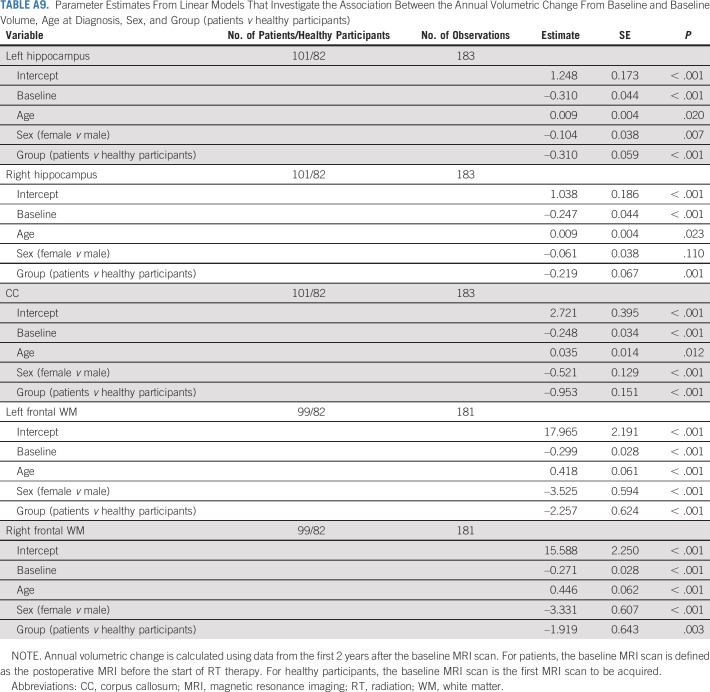
Parameter Estimates From Linear Models That Investigate the Association Between the Annual Volumetric Change From Baseline and Baseline Volume, Age at Diagnosis, Sex, and Group (patients *v* healthy participants)

## Data Availability

The data collected on this study cannot be made available to others.
